# Editorial: Implications of the inflammatory role of skin dendritic cells for health, disease and forensic practice

**DOI:** 10.3389/fmed.2024.1415354

**Published:** 2024-04-23

**Authors:** Montserrat Fernandez Guarino, Stefano Bacci

**Affiliations:** ^1^Dermatology Service, Hospital Ramón y Cajal, Instituto Ramón y Cajal de Investigación Sanitaria (IRYCIS), Madrid, Spain; ^2^Department of Biology, Research Unit of Histology and Embryology, University of Florence, Florence, Italy

**Keywords:** atopic dermatitis, dendritic cells, dermal Langerin, multiple sclerosis, kidney transplantation, SMRT and NCoR1

## Introduction

Dendritic cells (DC) are specialized antigen presenting cells abundant in peripheral tissues such as skin. DC are found in the dermis and in the epidermis (i.e., Langerhans cells) in the skin and they are bone marrow derived. It's known that skin DC migrate to the regional draining lymph node where they interact with naïve T cells to induce immune responses as acclaimed by the SALT concept proposed by *J. Wayne Streilein* in 1999.

This editorial summarizes the contributions to the Frontiers Research Topic “*Implications of the inflammatory role of skin dendritic cells for health, disease and forensic practice*” appearing in Frontiers in Medicine section Translational Medicine. To better address and clarify the issues relating to this research field, the Research Topic consists of a part dedicated to reviews on general or specific arguments in another containing research articles on specific Research Topics.

## Review on general argument

Multiple sclerosis (MS) is characterized by a compromised immune system that significantly impacts the spinal cord and brain. This condition is distinguished by a gradual inflammatory breakdown of the protective covering of nerve fibers, known as demyelination. Experimental autoimmune/allergic encephalomyelitis (EAE) models are the most often utilized animal models for studying multiple sclerosis (MS). This review aims to examine the interventions utilizing experimental autoimmune encephalomyelitis (EAE) models to assess the efficacy of exercise as a therapeutic approach for patients with MS. The objective is to offer a clear viewpoint for future research and the management of MS. Based on an analysis of the available findings, this literature review concludes that EAE is an appropriate animal model that can assist researchers in gaining a deeper comprehension of and developing treatments for MS (Parnow et al.).

### Research articles on specific Research Topics

Dendritic cells (DC) regulate the balance between inflammatory and tolerogenic responses in order to prevent immune-related damage. Nevertheless, the significance of coregulators in preserving this equilibrium remains uninvestigated. A recent study has demonstrated that NCoR1 is involved in the suppression of immunological tolerance in DC. In this study was discovered that the reduction of NCoR1 paralog SMRT (NCoR2) led to an increase in DC activation and in the expression of IL-6, IL-12, and IL-23 but a decrease of secretion of IL-10. The study of genomes and transcripts showed that IL-10 is regulated differently by SMRT and NCoR1. Depletion of SMRT suppresses the mTOR-STAT3-IL10 signaling pathway in DC by reducing the expression of NR4A1. In addition, the expression of Nfkbia and Socs3 was reduced in NCoR2 (Smrt) deficient DC, indicating an upregulation of inflammatory cytokine production. Furthermore, experiments conducted on mice demonstrated that the transfer of SMRT resulted in a decrease in DC, which in turn led to an increase in Th1/Th17 cells and a decrease in tumor size after injecting B16 melanoma. This was achieved by boosting the frequency of oncolytic CD8+ T-cells. Therefore, the analysis for the first time identified a fine switch that could be targeted to modulate inflammatory vs. tolerogenic programs in DC (Jha et al.).

Atopic dermatitis (AD) etiology has been linked to cutaneous DC. Nevertheless, it is unclear exactly what function each subset of DCs serves. In an AD animal model created by topical treatment of MC903 (calcitopriol), the purpose of this work was to examine the roles played by Langerhans cells (LC), resident dermal Langerin+ DC (r-Langerin+ DC), and newly infiltrated inflammatory dermal Langerin+ DC (i-Langerin+ DC). The findings showed that, after numerous injections of diphtheria toxin (DT), the reduction of i-Langerin+ DC in mLangerin-DTR (DTR) mice significantly reduced the production of thymic stromal lymphopoietin (TSLP) in lesions and improved skin inflammation. Unlike wild-type (WT) mice, the removal of LC or r-Langerin+ DC did not have a significant effect on the skin inflammation of hLangerin-DTA (DTA). Chimeric animals (DTR-WT) treated with PBS, which only depleted r-Langerin DC, exhibited inflammation that was comparable to that observed in WT mice. However, DT-treated DTR-WT chimeric mice, which had the depletion of BM-derived i-Langerin+ DC, showed much reduced skin inflammation compared to the control group. Moreover, TSLP aided in the maturation of i-Langerin+ DC and increased the expression of Langerin in BM-derived DC. In conclusion, the current investigation showed that the generation of local TSLP and AD development were dependent on the recently infiltrated inflammatory dermal Langerin+ DCs. Additionally, TSLP stimulated the creation of i-Langerin+ DC generated from bone marrow, which may sustain AD inflammation (Xiao et al.).

The development of donor-specific immune tolerance may enhance kidney transplant outcomes. There isn't a tolerance regimen available, nevertheless, for regular clinical application. Chimerism-based regimens have potential, but unsolved safety difficulties prevent them from being widely used. This study investigates the hypothesis that kidney transplant recipients who also receive donor bone marrow (BM) without myelosuppressive conditioning will experience transient chimerism and pro-tolerogenic immunomodulation as a result of therapy with polyclonal recipient regulatory T cells (Tregs) and anti-IL6R (tocilizumab). The trial's outcomes demonstrate that this method is practical, secure, and effective in producing transitory chimerism. If this combination cell therapy is effective, it could open up new avenues for organ transplant immunomodulation without the side effects of myelosuppressive recipient conditioning (Oberbauer et al.).

## Conclusions

Eminent scientists from many international schools, whose fields of study are focused on comprehending DC biology and immune responses, was called because of this Research Topic. Reviews have shown themselves to be punctual in their explanation and clarity, while research articles have put forth novel approaches and areas of study that will undoubtedly be explored further in the near future. Finally, the Research Topic raises the critical question of how vital the biology of DC is, supporting the notion that the problem is still far from resolved.

## Author contributions

SB: Conceptualization, Data curation, Formal analysis, Funding acquisition, Investigation, Project administration, Supervision, Validation, Visualization, Writing—original draft, Writing—review & editing. MF: Conceptualization, Data curation, Formal analysis, Funding acquisition, Investigation, Project administration, Supervision, Validation, Visualization, Writing—original draft, Writing—review & editing.

